# Factors affecting cataract surgical coverage and outcomes: a retrospective cross-sectional study of eye health systems in sub-Saharan Africa

**DOI:** 10.1186/s12886-015-0063-6

**Published:** 2015-06-30

**Authors:** Susan Lewallen, Elena Schmidt, Emma Jolley, Robert Lindfield, William H. Dean, Colin Cook, Wanjiku Mathenge, Paul Courtright

**Affiliations:** Kilimanjaro Centre for Community Ophthalmology International, Division of Ophthalmology, University of Cape Town, Cape Town, South Africa; Sightsavers, 35 Perrymount Road, Haywards Heath, RH16 3BW West Sussex, UK; International Centre for Eye Health, London School of Hygiene and Tropical Medicine (LSHTM), London, UK; Bristol Eye Hospital, Bristol, UK; Division of Ophthalmology, University of Cape Town, Cape Town, South Africa; Rwanda International Institute of Ophthalmology and Dr Agarwal’s Eye Hospital, Kigali, Rwanda

**Keywords:** Blindness, Cataract, Health systems

## Abstract

**Background:**

Recently there has been a great deal of new population based evidence on visual impairment generated in sub-Saharan Africa (SSA), thanks to the Rapid Assessment of Avoidable Blindness (RAAB) survey methodology. The survey provides information on the magnitude and causes of visual impairment for planning services and measuring their impact on eye health in administrative “districts” of 0.5–5 million people. The survey results describing the quantity and quality of cataract surgeries vary widely between study sites, often with no obvious explanation. The purpose of this study was to examine health system characteristics that may be associated with cataract surgical coverage and outcomes in SSA in order to better understand the determinants of reducing the burden of avoidable blindness due to cataract.

**Methods:**

This was a descriptive study using secondary and primary data. The outcome variables were collected from existing surveys. Data on potential district level predictor variables were collected through a semi-structured tool using routine data and key informants where appropriate. Once collected the data were coded and analysed using statistical methods including t-tests, ANOVA and the Kruskal-Wallis analysis of variance test.

**Results:**

Higher cataract surgical coverage was positively associated with having at least one fixed surgical facility in the area; availability of a dedicated operating theatre; the number of surgeons per million population; and having an eye department manager in the facility. Variables that were associated with better outcomes included having biometry and having an eye department manager in the facility.

**Conclusions:**

There are a number of health system factors at the district level that seem to be associated with both cataract surgical coverage and post-operative visual acuity outcomes. This study highlights the needs for better indicators and tools by which to measure and monitor the performance of eye health systems at the district level. It is unlikely that epidemiological data alone is sufficient for planning eye health services within a district and health managers and study coordinators need to consider collecting supplementary information in order to ensure appropriate planning can take place.

## Background

Cataract is the leading cause of blindness globally and in sub-Saharan Africa (SSA), where it is estimated to account for about half of all cases of blindness [1, 2]. An international initiative called Vision 2020: The Right to Sight, led by the World Health Organisation (WHO) and the International Agency for the Prevention of Blindness (IAPB) launched in 1999 was the first global effort to eliminate blindness from avoidable causes, including cataract which can only be treated surgically [3].

Cataract surgical coverage (CSC) is the indicator used to estimate the extent to which a population’s cataract surgical needs have been met. CSC is expressed as the proportion of visually impaired individuals with cataract who require surgery who actually receive it. Ideally this would be close to 100 %, however the CSC in many SSA settings remains consistently low with some surveys reporting less than one person in ten receiving the surgery they require [4]. The barriers to achieving a higher CSC are multiple and may include a number of factors on both the health system and patient side. These include insufficient surgeons, or auxiliary ophthalmic staff; inadequate facilities in which to operate; insufficient equipment, supplies or other resources necessary for surgery; inefficient diagnostic or referral services to identify people with cataracts; or unwillingness of the population to attend services. Evidence also shows that CSC is lower among women than men, indicating additional barriers to access associated with gender [5].

While the CSC indicator informs us about the quantity of surgeries produced in a year, it is also important to measure the quality of the surgeries provided to the population. Although quality of life and patient experience measures are vital dimensions of quality, they are difficult to measure and so post-operative visual acuity (VA) is considered an informative indicator on surgical quality. The WHO recommends that good post-operative outcomes should be seen in at least 80 % of operated eyes and that no more than 5 % should have a poor outcome [6]. A “good” outcome is defined as a post-operative presenting VA of 6/18 or better while a “poor” outcome is defined as presenting VA worse than 6/60 [7]. Unfortunately cataract surgical outcomes in many SSA settings do not meet the WHO recommended level, and recent population-based studies report good outcomes in as few as 30 % of cases [8]. Factors that may decrease the likelihood of good outcomes include surgical complications, ocular co-morbidities, uncorrected refractive error and long term complications. Skilled surgeons with access to appropriate resources should be able to prevent or mitigate against these factors in order to promote the best possible outcomes for cataract patients.

CSC can only be measured through population based studies. Post-operative VA outcomes should be routinely captured through the health monitoring information systems, both a day or two after surgery and again after 40 days when VA should have stabilised. Unfortunately this information, particularly at 40 days, is rarely captured on a regular basis, thus longer term VA outcomes are often measured through population based surveys, although there remain other challenges with this form of monitoring.

The standardised survey methodology known as RAAB (rapid assessment of avoidable blindness) was developed in order to systematically assess the magnitude and causes of blindness in the most affected age groups, i.e., the population aged over 50. In addition it provides an estimate of the cataract surgical coverage and the outcomes of cataract surgery [9]. The RAAB was developed in order to provide information for both planning services and measuring their impact at a “district” level (or in a population of between 500,000 and five million people) relatively quickly and with minimal resources. While these surveys attempt to address individual-level barriers to accessing cataract services, they do not provide information on the supply-side or health system factors that can greatly influence both the quantity and quality of cataract surgeries.

The health system is described by WHO as all the organizations, people and actions whose primary intent is to promote, restore or maintain health [10]. Despite regional, national and local differences, many health systems share similar features and so a number of frameworks exist to describe how a generic health system functions. The most commonly referenced is the WHO’s concept of six building blocks that describe the system as separate, but interdependent, components: human resources; technology & consumables; governance; finance; health information; and service delivery [11].

The VISION 2020 initiative recommends that eye health services be organised at the micro, or district level where budgetary decisions are made, facilities and specialist staff are based, equipment and supplies are procured and distributed, and health information is collected. Decisions and actions taken at this level therefore have a great influence over the quantity and quality of services provided to the catchment population. However, it is unclear what combination of district level factors may promote increased quantity and quality of eye health services, including cataract surgeries, and thus where district decision makers should focus their limited resources.

Increasingly, as more RAABs are undertaken in SSA the knowledge base on the magnitude and causes of visual impairment, especially due to cataract grows. The purpose of this study was to examine “district” health system factors that may be associated with CSC and VA outcomes after cataract surgery in SSA in order to better understand the determinants of achieving the Vision 2020 targets. We limited the study to SSA as it is unlikely that health systems in other low or middle income settings such as Asia or Latin America would be similar enough to allow for meaningful comparison.

## Methods

In total 27 RAABs conducted in SSA in 2005–2012, excluding the WHO Eastern Mediterranean Region were identified by contacting known RAAB trainers. The RAAB from Cape Town, South Africa was excluded, as we did not consider it to be representative of SSA. Permission was obtained to use both published and unpublished survey data from the principal investigators of each survey. Ethical clearance for the study was granted by the Human Research Ethics Committee of the University of Cape Town.

### Measuring CSC and VA outcomes

The dependent variables used in the analysis were CSC and VA outcome after surgery, both of which are calculated in a standard fashion by the RAAB software [9]. CSC is usually reported both by eye and by person and at three different levels of presenting VA (i.e., <3/60 in the better eye; <6/60–3/60 in the better eye; and <6/18–6/60 in the better eye). We used CSC by eye at the 6/60 level for all analyses because this variable was available for all RAABs included in the study; we also tested and found a strong correlation among the various measures of CSC reported in the RAABs.

VA outcomes measured in RAABs are reported as the percentage of all operated eyes, regardless of the type of operation, with a good outcome, poor outcome, and borderline outcome (VA worse than 6/18 but better or equal to 6/60). As a single overall measure of outcome we used a composite (percentage difference) calculated by subtracting the percent of poor outcomes from the percent of good outcomes. Outcomes were used for all operated eyes in the sample for each site, regardless of when the operation was done. Presenting vision was used, rather than pinhole.

### Collecting data on health systems

Information on health system factors that may predict CSC and VA outcomes was collected using semi-structured interviews with purposefully selected informants in each RAAB site. The respondents (one per site) were nominated by the local authorities in each RAAB area and included most often the ophthalmologist at the main facility, but also NGO program directors, and national eye coordinators. Some of the responders were involved in the respective RAABs; our questions were related only to the health systems and not the survey itself.

The tool used to collect data was based on the six health system “building blocks” identified in the WHO Health system framework [11]. These include (i) human resources, (ii) technology & consumables, (iii) governance, (iv) finance, (v) health information, and (vi) service delivery. Based on the characteristics of eye health systems reported in the literature and our own experience in SSA, within each “building block” we identified aspects of eye health service delivery, which may be associated with CSC and VA outcomes. These included the number and type of eye health facilities in the district; availability of outreach services; availability and functionality of equipment; the number and type of eye health personnel; and physical and financial access to services by patients. A semi-structured questionnaire asking about these aspects of the system in the five years preceding the RAAB was developed and piloted in two sites. Five years was selected because services over this duration would be expected to have some impact on CSC and VA outcomes and because recall was expected to decrease with more years. Semi-structured interviews were conducted face-to-face and over the phone by three researchers; the average time of the interview was 30 min. Responses to both closed and open ended questions were written up in a narrative form and emailed to the interviewee for corrections, clarifications and confirmation. Follow up emails and phone calls were used to clarify points of uncertainty. The informants were told about the purpose of the study, however since the explanatory variables were not fully defined until after the interviews, the likelihood of systematic bias in their responses is quite low. For some participants, several years may have passed since the time period in question which may have resulted in some errors in their recollections.

### Data analysis

Data analysis was done in two stages. First, a batch of 10 anonymised interviews were read by a panel of seven researchers, some of whom were involved in design of the questionnaire and some of whom were not; the panel was asked to develop a system of coding and rating for all variables that in their view could be associated with CSC and VA outcomes. An interactive process was used to cross- check the system of coding and agree the final system of rating (Table 1).

**Table 1:** Potential explanatory variables investigated

Indicator	Description
Number of fixed facilities/million population	We examined this variable in 2 different ways: first as a categorical variable: between 0 and 1; between 1 and 2; 2 or more. We also examined it as a dichotomous variable: no facilities; any facilities.
Presence of a dedicated eye theatre in at least one of the facilities	Dichotomous variable: Yes; No.
Number of surgeons/year/million population	To calculate number of surgeons, all surgeons stationed full time in the district during the 5 years were included; their contribution was weighted by the number of years (or fraction) they were there and this was averaged over 5 years.
Presence of functioning biometry in at least one facility	Dichotomous variable: Yes; No.
Surgical outreach: did any take place within the district?	Dichotomous variable: Yes; No.
Was there a manager for the site?	Dichotomous variable: Yes; No.
Was there an NGO hospital in the district?	Dichotomous variable: Yes; No.
Was there an eye NGO supporting activities within the district?	Dichotomous variable: Yes; No.
GDP per capita	Gross domestic product per capita in US$ by country for closest year of survey [16]
Health expenditure per capita	Health expenditure per capita in US$ by country for closest year of survey [17]
Population density	District population/district area in square meters

Three additional reviewers, none of whom was involved in design of the questionnaire or interviews were then asked to read all the narrative descriptions and apply the coding system to each. All narratives were rendered anonymous by removing names of hospitals and administrative area labels such as province or district; surveys that were for a whole country were identified as such. Information on population size and density was provided to give a better sense of the context in which services were delivered. Definitions and the purpose of asking about the variable were included to inform their use and the option of “cannot tell” was offered for most of the variables. The results were compared across the reviewers and disagreements were reconciled, referring back to the narratives, contacting the interviewee or using “cannot tell” option.

Statistical analysis was done using Stata 11 (StataCorp. 2009. *Stata Statistical Software: Release 11*. College Station, TX: StataCorp LP.). Six independent variables were excluded from the analysis because the data were incomplete (many respondents could not answer the question) or responses were too heterogeneous to include in the analysis. These included questions intended to describe surgical outreach; patient fees and transport; and support personnel in the fixed facilities. Some facility-based data were difficult to interpret in the sites with multiple facilities where different practices existed. A decision was made to analyse such variables as dichotomous, where the characteristic was present if it applied to at least one facility in the site. In total eight independent eye health system related variables were tested for an association with CSC and six variables for an association with VA outcomes. The number of facilities and eye health personnel were converted to ratios per million population. Additional non health system data, including population density in the district, national gross domestic product (GDP) and total health expenditures were also collected and included in the analysis to test the effect of these factors.

## Results

In total 24 RAAB sites were included in the analysis since despite multiple attempts over a year no knowledgeable responsive informant could be identified in two of the 26 sites originally identified. There were substantial variations in both CSC (at <6/60 by eye) and VA outcomes (percentage difference of good and poor outcomes) between the sites. For CSC, there was a fivefold difference between the lowest CSC (9 %) and the highest CSC (55 %); the mean was 30.4 % (SD 11.6) and the median was 29 %. Regarding cataract surgical outcomes, in 7 (29 %) sites poor outcomes outnumbered good outcomes, resulting in a negative percentage difference; the overall range was from −24.4 % to +53.5 %. The mean outcome difference was 13.6 % (SD 22.8) and the median was 17.5 %. There was no correlation (coefficient = 0.07 SE = 0.11, *p* = 0.50) between CSC and cataract surgical outcome.

Regarding eye health system factors, 6 sites had no fixed eye care facility or local surgical staff, 7 had a single fixed facility (one was a national RAAB) and 11 sites had more than one fixed facility (3 of these were national RAABs and 8 were “district” RAABs). The mean number of facilities/million population was 1.8 (SD 1.6), the median was 0.71 and the range was 0–6.1. Regarding surgeons, 6 sites had none and the other sites varied from 1.4 to 19 surgeons. (Fractions reflect the fact that some surgeons were not present for the entire 5 year period of interest.) The mean number of surgeons per million population was 3.4 (SD 4.1), the median was 2.5 and the range was 0–19. Only four sites with a fixed facility had an eye department manager. Over 87 % of sites (21 out of 24) reported that they provided surgical outreach but only a third of these (8 out of 21) used surgeons from within the area; in other sites outreach was done by surgeons from visiting teams. One third of the sites with a fixed facility (6 out of 18) had no dedicated operating theatre and two thirds (10 out of 18) did not have a functional biometry. NGO hospitals were present in 29 % (7 out of 24) of the sites.

The results of testing variables expected to predict CSC (by eye at 6/60) are shown in Table 2. Four of the eight assessed factors had a statistically significant association with CSC. Higher CSC was positively associated with having at least one fixed surgical facility in the area (*p* = 0.01); availability of a dedicated operating theatre (*p* = 0.03); the number of surgeons per million population (*p* = 0.026) and having an eye department manager in the facility (*p* = 0.003).

**Table 2:** Association of factors with cataract surgical coverage (<6/60 by eye)

Factor (number with data for analysis)		Mean CSC (SD)	*P* (test type)
Number of fixed facilities/million population (N = 24)			
	0 (6)	22.0 % (8.0)	0.14 (anova)*
	more than 0 but <1 (8)	30.9 % (10.9)	
	1, but less than 2 (5)	32.0 % (7.3)	
	2 or more (10)	38.0 % (15.7)	
	None	22.0 % (8.0)	0.01 (*t*-test)
	More than zero	33.2 % (11.4)	
Presence of a dedicated eye theatre in at least one of the facilities (N = 18, NA = 6)			
	No (6)	26.4 (6.4)	0.03 (*t*-test)
			0.07 (KW)
	Yes (12)	36.6 (11. 9)	
Number of surgeons/year/million population (N = 24)			
	0 (6)	22.0 (8.0)	0.026 (anova)**
	0.3–1.4 (6)	27.6 (7.5)	
	1.5–2.7 (6)	33.4 (10.6)	
	2.8–6.1 (6)	38.6 (14.1)	
Presence of functioning biometry in at least one facility (N = 17, NA = 6, CT = 1)			
	No (10)	31.7 % (9.2)	0.25 (*t*-test)
	Yes (7)	35.8 % (15.0)	
Surgical outreach: did any take place within the district? (N = 24)			
	No (3)	38.3 (14.9)	0.11 (*t*-test)
	Yes (21)	29.3 (11.0)	
Was there a manager for the site? (n = 14, NA = 6, CT = 4)			
	No (10)	27.7 % (9.6)	0.003 (*t*-test)
	Yes (4)	46.6 % (8.5)	
Was there an NGO hospital in the district? (N = 24)			
	No (17)	28.7 % (9.6)	0.14 (t = test)
	Yes (7)	34.5 % (15.4)	

*KW* Kruskal-Wallis, *NA* not applicable, *CT* cannot tell

* Treated as a continuous variable the regression coefficient = 2.9 (SE 1.7), *p* = 0.10

** Treated as a continuous variable the regression coefficient = 4.2 (SE 1.2), *p* = 0.002

Table 3 shows the results of testing variables expected to be associated with VA outcomes after the surgery. Variables that were associated with better outcomes included having biometry (*p* = 0.005) and having an eye department manager in the facility (*p* = 0.02).

**Table 3:** Association of factors with cataract surgical outcome

Factor (number with data for analysis)		Mean cataract surgical outcome (SD)	*P* (test type)
Presence of functioning biometry in at least one FF (N = 18, NA = 6)			
	No	0.4 (18.0)	0.005 (KW)
	Yes	27.7 (12.8)	
Surgical outreach: did any take place within the district? (N = 24)			
	No	−3.8 (26.5)	0.15 (KW)
	Yes	16.1 (21.8)	
If yes to previous question, from where did surgeons come? (N = 21, NA = 3)			
	Within district only:	4.6 (18.3)	0.14 (KW)
	Outside district:	21.9 (21.6)	
Was there a manager for the site? (n = 14, NA = 6, CT = 4)			
	No	1.9 (20.1)	0.02 (KW)
	Yes	31.6 (9.2)	
Was there an NGO hospital in the district? (N = 24)			
	No	10.6 (22.6)	0.24 (KW)
	Yes	21.1 (23.2)	
Was there an eye NGO supporting activities within the district? (N = 24)			
	No	21.8 (29.0)	0.13 (KW)
	Yes	10.3 (19.7)	

*KW* Kruskal-Wallis, *NA* not applicable, *CT* cannot tell

None of the three non-health system indicators predicted coverage or cataract surgical outcomes.

## Discussion

The goal of the study was to explore reasons for variable CSC and cataract surgical outcomes, both of which showed huge variations across the 24 settings. Cataract service provision in the examined settings varied from sites with no surgical facility (most likely surveyed before an NGO anticipated starting work) to sites with long standing services enjoying substantial external support. Although the extent to which these sites are representative of eye care in Africa is debatable, overall, it is clear that many settings included in this study require significant strengthening of local eye care services to achieve improved availability of surgery and quality of surgical outcomes.

The finding that higher CSC is associated with more surgeons per million population comes as no surprise; similar conclusions were made in a recent study examining national cataract surgical rates and the number of surgeons available nationally in SSA countries [12]. Our findings however suggest that the relationship may be non-linear; the site with the third lowest CSC (16.2 %) had a higher than average ratio (2.8 per million) of surgeons per population (data not shown). The Vision 2020 initiative suggested a target of 4 ophthalmologists per million population [13] however this number may be more than is needed in some districts and insufficient in others. Other studies in SSA have demonstrated that surgeon productivity is highly variable [14, 15].

Availability of a fixed facility with a dedicated operating theatre was associated with about 10 percentage points higher CSC, however this was not statistically significant. The lack of significance may be due to small sample size. Cataract coverage is a function of complex interactions between service supply and patient demand; the latter being often limited by high surgery fees, lack of transport or lack of information about the surgery and its benefits.

It is also interesting that the sites where at least one of the facilities had a dedicated manager had significantly higher CSC; however these numbered only four and may reflect the overall impact of a better organized and supported eye care rather than the presence of a manager per se. In this study we did not have data on facility-specific resources and management and were not able to test this hypothesis. In addition, the number of observations is too small for meaningful multivariable analysis. A better understanding of the relationship between the level of external support to eye care and CSC should be a priority for future research.

Cataract surgical outcomes were associated with the presence of biometry and a manager. The positive association of biometry with better outcomes reiterates the need for routine use of biometry in all settings. However, both findings are limited by the fact that only one facility in the site had to meet the criterion, highlighting again the difficulties of describing health systems when there are multiple providers.

It is commonly believed that if the quality of surgery is good, the coverage will increase. The lack of an association between the CSC and cataract surgical outcomes in these 24 sites may be due to a small number of observations or it may reflect the fact that coverage is a complex issue, as noted above, and includes factors besides outcomes.

It is interesting to note that, even in settings with no fixed facilities or surgeons that some people (about 22 % of eyes with <6/60 cataract) have received surgery. Also, the mean value of surgical outcomes in the 6 districts without fixed facilities was 20.8, slightly better than the mean value of 11.3 in the other 18 settings. Whether people travel outside the district to get services or surgeons from outside come and provide services could not be determined from the RAAB reports available but would be useful information to inform planning.

The process for identifying and defining the potential factors for this analysis was more challenging than expected. Few of the sites had records or reports describing the service provision. Many respondents had limited knowledge of the situation when there were multiple fixed facilities. With the paucity of services that is generally agreed to be a problem in SSA, we expected that most of the “districts” which have had RAABs would have no more than one fixed facility offering cataract surgical services. However, in only seven of the 18 settings with fixed facilities was this the case. In these settings all the variables we developed were applicable and unambiguous. In the other 11 settings (three of which were national RAABs) some information collected had to be considered as “available in one or more facility”; and our conclusions on the association between CSC or VA outcomes and health system characteristics are weaker because of this.

Information on variables that were not specific for a facility were easier to collect; these included number of facilities, surgeons, presence of an NGO hospital in the district, and support by an international eye care NGO to district services, although the latter requires a more precise definition to differentiate the extent of support, which can be highly variable. It is popular for small NGOs to make short term excursions once a year or so to provide cataract surgery but this is not the same as major sustained support to a government or NGO eye program.

Capturing information of the eye health services over a five year period is difficult because services can be very fluid, depending upon support, staffing, and other factors. We made effort to limit data collection to the five year period before the RAAB and used fractions as necessary, for example to calculate the number of surgeons present at the site. Strengthening district level information systems and systematic recording of the key parameters of the health service delivery available in the site, can improve the accuracy of data and mitigate the impact of recall bias.

## Conclusion

Even with a number of limitations, there are important lessons learned from the research.

First, this study illustrates that fact that health systems are complex and interrelated; breaking them into measurable components can be challenging. The “six building blocks” suggested by the WHO are a useful macro tool but we need more specific measurable indicators as well; in this study we tried to elucidate what these might be for providing eye care.

Second, while the stated goal for undertaking a RAAB is to generate data for the purpose of planning, it appears that this is not often done. The fact that in two RAAB sites there was no one available in either the government or the NGO sector or provide basic information on eye health services highlights this and begs the question of why surveys were done there. Usefulness of the RAAB could be enhanced by collecting information such as the predictive factors included in this study at the time of the RAAB; this would help planners interpret the findings from the RAAB as well as better determine what changes are needed to improve cataract (and other) services. The options for asking operated patients where they had surgery in the current RAAB tool should be considered and modified in each setting to provide locally useful information.

Third, the assumption that national level GDP and health expenditure predicts CSC is not demonstrated in this study. Findings from two or more RAABs within the same country have shown widely divergent results, suggesting that local efforts at building eye health systems are more important than nationwide approaches.

In summary, findings from this study illustrate a few of the many contributors to high CSC and good cataract surgical outcomes and suggest that efforts to increase both CSC and outcomes in sub-Saharan Africa is possible but requires addressing many different components of the local eye health system at the same time.

## Abbreviations

CSC: Cataract surgical coverage
GDP: Gross domestic product
HMIS: Health management information system
IAPB: International Agency for the Prevention of Blindness
NGO: Non-governmental organisation
RAAB: Rapid assessment of avoidable blindness
SD: Standard deviation
SE: Standard error
SSA: Sub-Saharan Africa
VA: Visual acuity
WHO: World Health Organisation

## References

[1] Resnikoff S, Pascolini D, Etya’ale D, Kocur I, Pararajasegaram R, Pokharel GP (2004). Global data on visual impairment in the year 2002. Bull WHO.

[2] Pascolini D, Mariotti SP (2012). Global estimates of visual impairment: 2010. Br J Ophthalmol.

[3] World Health Organization. Universal eye health: a global action plan 2014–2019. World Health Organization. 2013. http://www.who.int/blindness/actionplan/en/. Accessed October 1, 2014.

[4] Rapid assessment of avoidable blindness repository. International Centre for Eye Health, London School of Hygiene & Tropical Medicine. 8 % Nampula, Mozambique. 2011. http://www.raabdata.info/repository/. Accessed October 1, 2014.

[5] Lewallen S, Mousa A, Bassett K, Courtright P (2009). Cataract surgical coverage remains lower in women. Br J Ophthalmol.

[6] World Health Organisation. Informal consultation on analysis of blindness prevention outcomes. Geneva: World Health Organization; 1998.

[7] Limburg H, Foster A, Gilbert C, Johnson GJ, Kyndt M (2005). Routine monitoring of visual outcome of cataract surgery. Part 1: Development of an instrument. Br J Ophthalmol.

[8] Lindfield R, Vishwanath K, Ngounou R, Khanna RC (2012). The challenge of improving outcome of cataract surgery in low and middle income countries. Indian J Ophthalmol.

[9] Kuper H, Polack S, Limburg H (2006). Rapid assessment of avoidable blindness. Community Eye Health.

[10] World Health Organisation (2000). World health report 2000.

[11] World Health Organisation (2007). Everybody’s business — strengthening health systems to improve health outcomes. WHO’s framework for action.

[12] Palmer J, Chinanayi F, Gilbert A, Pillay D, Fox S, Jaggernath J (2014). Mapping human resources for eye health in 21 countries of sub-Saharan Africa: current progress towards VISION 2020. Hum Resour Health.

[13] World Health Organisation. V2020: global initiative for the elimination of avoidable blindness: action plan 2006 to 2011. World Health Organisation; 2007. http://www.who.int/blindness/Vision2020_report.pdf. Accessed October 1, 2014.

[14] Courtright P, Ndegwa L, Msosa J, Banzi J (2007). Use of our existing eye care human resources: assessment of the productivity of cataract surgeons trained in eastern Africa. Arch Ophthalmol.

[15] Habtamu E, Eshete Z, Burton MJ (2013). Cataract surgery in Southern Ethiopia: distribution, rates and determinants of service provision. BMC Health Serv Res.

[16] GDP per capita data. The World Bank. 2014. http://data.worldbank.org/indicator/NY.GDP.PCAP.CD?order=wbapi_data_value_2013+wbapi_data_value+wbapi_data_value-last& sort=asc. Accessed October 1, 2014.

[17] Health expenditure per capita data. The World Bank data. 2014 http://data.worldbank.org/indicator/SH.XPD.PCAP. Accessed October 1, 2014.

